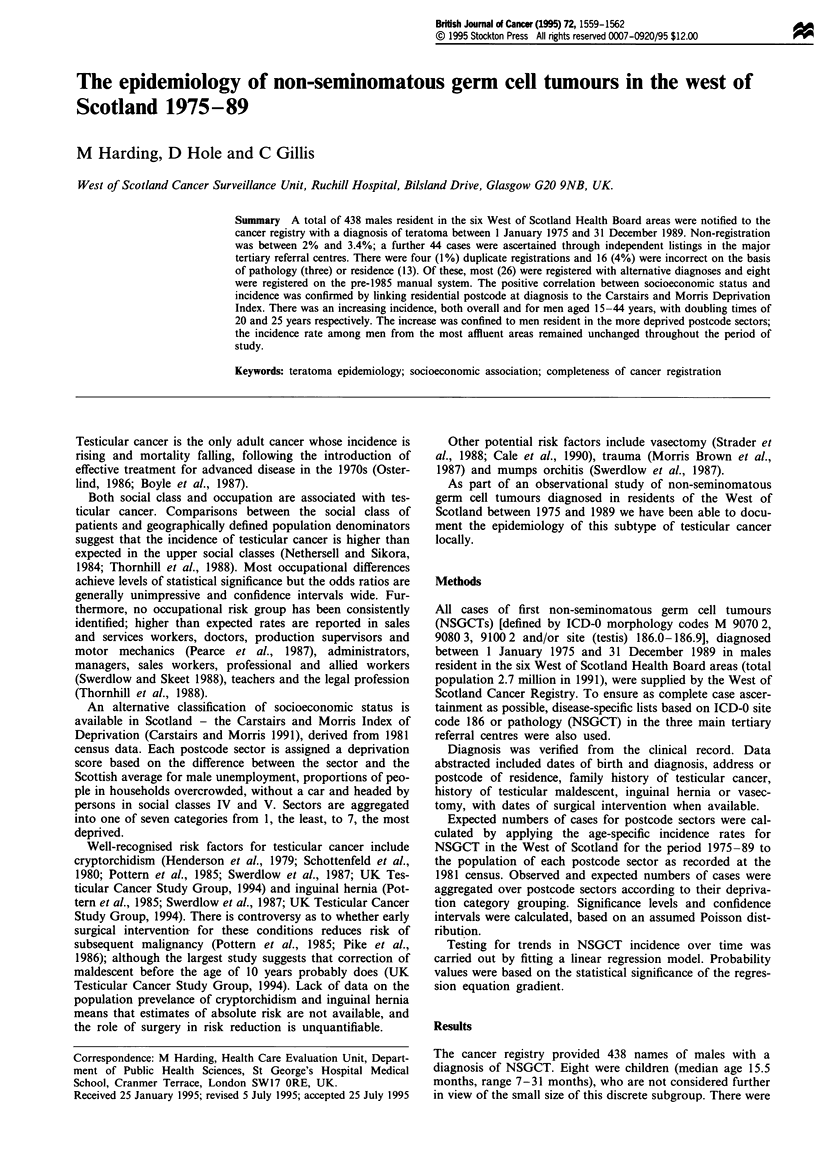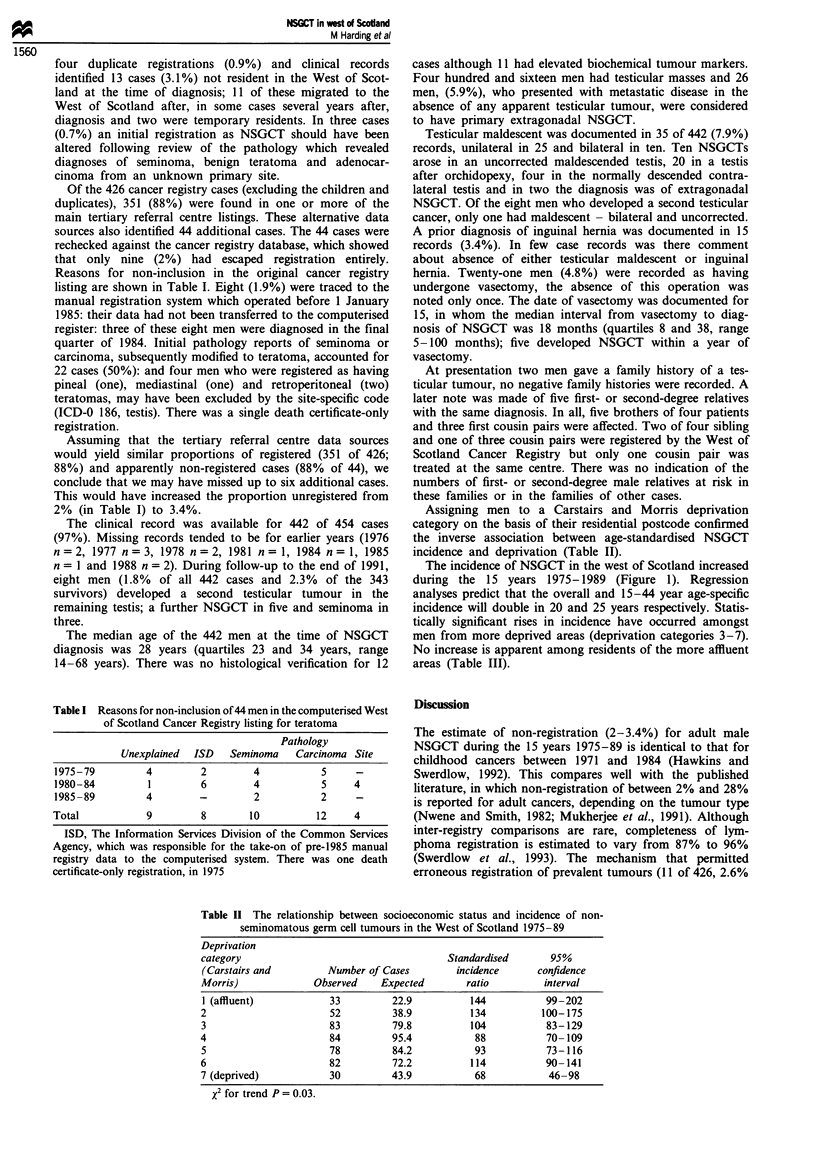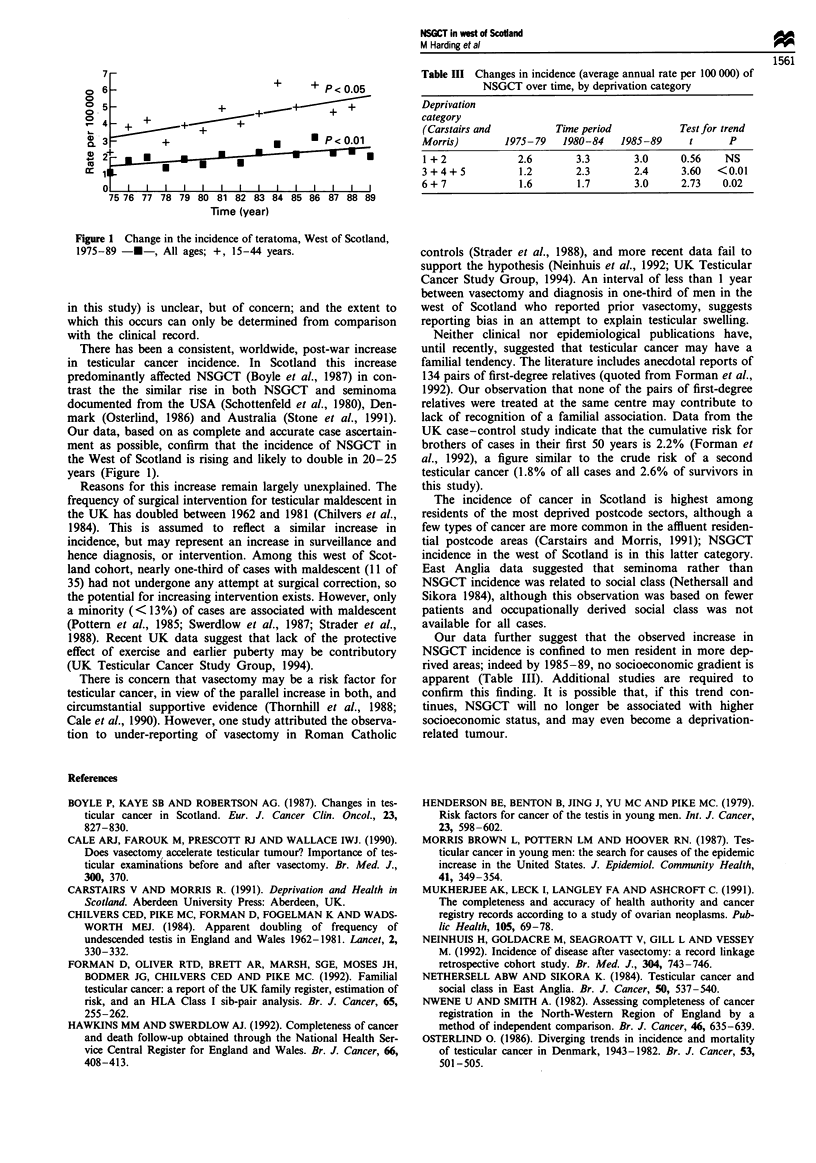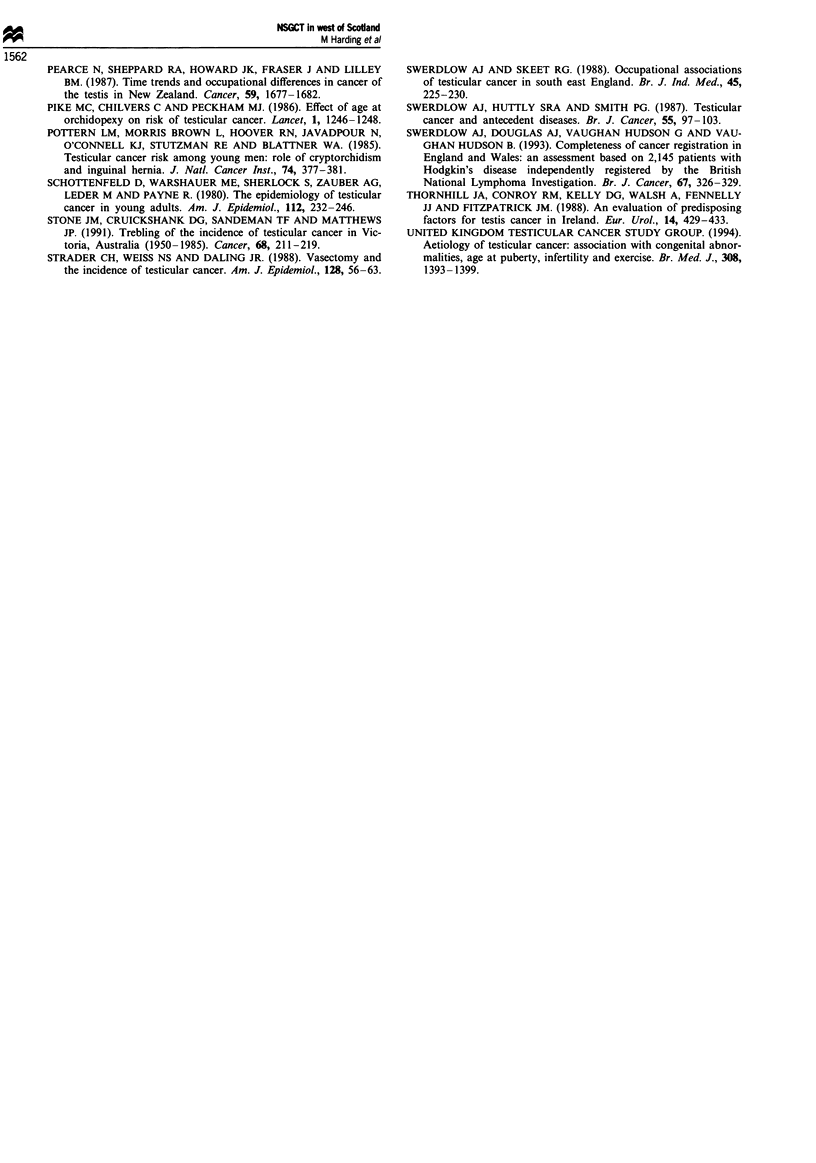# The epidemiology of non-seminomatous germ cell tumours in the west of Scotland 1975-89.

**DOI:** 10.1038/bjc.1995.548

**Published:** 1995-12

**Authors:** M. Harding, D. Hole, C. Gillis

**Affiliations:** West of Scotland Cancer Surveillance Unit, Ruchill Hospital, Glasgow, UK.

## Abstract

A total of 438 males resident in the six West of Scotland Health Board areas were notified to the cancer registry with a diagnosis of teratoma between 1 January 1975 and 31 December 1989. Non-registration was between 2% and 3.4%; a further 44 cases were ascertained through independent listings in the major tertiary referral centres. There were four (1%) duplicate registrations and 16 (4%) were incorrect on the basis of pathology (three) or residence (13). Of these, most (26) were registered with alternative diagnoses and eight were registered on the pre-1985 manual system. The positive correlation between socioeconomic status and incidence was confirmed by linking residential postcode at diagnosis to the Carstairs and Morris Deprivation Index. There was an increasing incidence, both overall and for men aged 15-44 years, with doubling times of 20 and 25 years respectively. The increase was confined to men resident in the more deprived postcode sectors; the incidence rate among men from the most affluent areas remained unchanged throughout the period of study.


British Journal of Cancer (1995) 72, 1559-1562
© 1995 Stockton Press All rights reserved 0007-0920/95 $12.00

### The epidemiology of non-seminomatous germ cell tumours in the west of Scotland 1975-89

M Harding, D Hole and C Gillis

*West of Scotland Cancer Surveillance Unit, Ruchill Hospital, Bilsland Drive, Glasgow G20 9NB, UK.*

**Summary** A total of 438 males resident in the six West of Scotland Health Board areas were notified to the cancer registry with a diagnosis of teratoma between 1 January 1975 and 31 December 1989. Non-registration was between 2% and 3.4%; a further 44 cases were ascertained through independent listings in the major tertiary referral centres. There were four (1%) duplicate registrations and 16 (4%) were incorrect on the basis of pathology (three) or residence (13). Of these, most (26) were registered with alternative diagnoses and eight were registered on the pre-1985 manual system. The positive correlation between socioeconomic status and incidence was confirmed by linking residential postcode at diagnosis to the Carstairs and Morris Deprivation Index. There was an increasing incidence, both overall and for men aged 15-44 years, with doubling times of 20 and 25 years respectively. The increase was confined to men resident in the more deprived postcode sectors; the incidence rate among men from the most affluent areas remained unchanged throughout the period of study.

**Keywords:** teratoma epidemiology; socioeconomic association; completeness of cancer registration

Testicular cancer is the only adult cancer whose incidence is rising and mortality falling, following the introduction of effective treatment for advanced disease in the 1970s (Osterlind, 1986; Boyle *et al.*, 1987).

Both social class and occupation are associated with testicular cancer. Comparisons between the social class of patients and geographically defined population denominators suggest that the incidence of testicular cancer is higher than expected in the upper social classes (Nethersell and Sikora, 1984; Thornhill *et al.*, 1988). Most occupational differences achieve levels of statistical significance but the odds ratios are generally unimpressive and confidence intervals wide. Furthermore, no occupational risk group has been consistently identified; higher than expected rates are reported in sales and services workers, doctors, production supervisors and motor mechanics (Pearce *et al.*, 1987), administrators, managers, sales workers, professional and allied workers (Swerdlow and Skeet 1988), teachers and the legal profession (Thornhill *et al.*, 1988).

An alternative classification of socioeconomic status is available in Scotland - the Carstairs and Morris Index of Deprivation (Carstairs and Morris 1991), derived from 1981 census data. Each postcode sector is assigned a deprivation score based on the difference between the sector and the Scottish average for male unemployment, proportions of people in households overcrowded, without a car and headed by persons in social classes IV and V. Sectors are aggregated into one of seven categories from 1, the least, to 7, the most deprived.

Well-recognised risk factors for testicular cancer include cryptorchidism (Henderson *et al.*, 1979; Schottenfeld *et al.*, 1980; Pottern *et al.*, 1985; Swerdlow *et al.*, 1987; UK Testicular Cancer Study Group, 1994) and inguinal hernia (Pottern *et al.*, 1985; Swerdlow *et al.*, 1987; UK Testicular Cancer Study Group, 1994). There is controversy as to whether early surgical intervention for these conditions reduces risk of subsequent malignancy (Pottern *et al.*, 1985; Pike *et al.*, 1986); although the largest study suggests that correction of maldescent before the age of 10 years probably does (UK Testicular Cancer Study Group, 1994). Lack of data on the population prevelance of cryptorchidism and inguinal hernia means that estimates of absolute risk are not available, and the role of surgery in risk reduction is unquantifiable.

Correspondence: M Harding, Health Care Evaluation Unit, Department of Public Health Sciences, St George's Hospital Medical School, Cranmer Terrace, London SW17 0RE, UK.
Received 25 January 1995; revised 5 July 1995; accepted 25 July 1995

Other potential risk factors include vasectomy (Strader *et al.*, 1988; Cale *et al.*, 1990), trauma (Morris Brown *et al.*, 1987) and mumps orchitis (Swerdlow *et al.*, 1987).

As part of an observational study of non-seminomatous germ cell tumours diagnosed in residents of the West of Scotland between 1975 and 1989 we have been able to document the epidemiology of this subtype of testicular cancer locally.

### Methods

All cases of first non-seminomatous germ cell tumours (NSGCTs) [defined by ICD-0 morphology codes M 9070 2, 9080 3, 9100 2 and/or site (testis) 186.0-186.9], diagnosed between 1 January 1975 and 31 December 1989 in males resident in the six West of Scotland Health Board areas (total population 2.7 million in 1991), were supplied by the West of Scotland Cancer Registry. To ensure as complete case ascertainment as possible, disease-specific lists based on ICD-0 site code 186 or pathology (NSGCT) in the three main tertiary referral centres were also used.

Diagnosis was verified from the clinical record. Data abstracted included dates of birth and diagnosis, address or postcode of residence, family history of testicular cancer, history of testicular maldescent, inguinal hernia or vasectomy, with dates of surgical intervention when available.

Expected numbers of cases for postcode sectors were calculated by applying the age-specific incidence rates for NSGCT in the West of Scotland for the period 1975-89 to the population of each postcode sector as recorded at the 1981 census. Observed and expected numbers of cases were aggregated over postcode sectors according to their deprivation category grouping. Significance levels and confidence intervals were calculated, based on an assumed Poisson distribution.

Testing for trends in NSGCT incidence over time was carried out by fitting a linear regression model. Probability values were based on the statistical significance of the regression equation gradient.

### Results

The cancer registry provided 438 names of males with a diagnosis of NSGCT. Eight were children (median age 15.5 months, range 7-31 months), who are not considered further in view of the small size of this discrete subgroup. There were four duplicate registrations (0.9%) and clinical records identified 13 cases (3.1%) not resident in the West of Scotland at the time of diagnosis; 11 of these migrated to the West of Scotland after, in some cases several years after, diagnosis and two were temporary residents. In three cases (0.7%) an initial registration as NSGCT should have been altered following review of the pathology which revealed diagnoses of seminoma, benign teratoma and adenocarcinoma from an unknown primary site.

Of the 426 cancer registry cases (excluding the children and duplicates), 351 (88%) were found in one or more of the main tertiary referral centre listings. These alternative data sources also identified 44 additional cases. The 44 cases were rechecked against the cancer registry database, which showed that only nine (2%) had escaped registration entirely. Reasons for non-inclusion in the original cancer registry listing are shown in Table I. Eight (1.9%) were traced to the manual registration system which operated before 1 January 1985: their data had not been transferred to the computerised register: three of these eight men were diagnosed in the final quarter of 1984. Initial pathology reports of seminoma or carcinoma, subsequently modified to teratoma, accounted for 22 cases (50%): and four men who were registered as having pineal (one), mediastinal (one) and retroperitoneal (two) teratomas, may have been excluded by the site-specific code (ICD-0 186, testis). There was a single death certificate-only registration.

Assuming that the tertiary referral centre data sources would yield similar proportions of registered (351 of 426; 88%) and apparently non-registered cases (88% of 44), we conclude that we may have missed up to six additional cases. This would have increased the proportion unregistered from 2% (in Table I) to 3.4%.

The clinical record was available for 442 of 454 cases (97%). Missing records tended to be for earlier years (1976 $n = 2$, 1977 $n = 3$, 1978 $n = 2$, 1981 $n = 1$, 1984 $n = 1$, 1985 $n = 1$ and 1988 $n = 2$). During follow-up to the end of 1991, eight men (1.8% of all 442 cases and 2.3% of the 343 survivors) developed a second testicular tumour in the remaining testis; a further NSGCT in five and seminoma in three.

The median age of the 442 men at the time of NSGCT diagnosis was 28 years (quartiles 23 and 34 years, range 14–68 years). There was no histological verification for 12 cases although 11 had elevated biochemical tumour markers. Four hundred and sixteen men had testicular masses and 26 men, (5.9%), who presented with metastatic disease in the absence of any apparent testicular tumour, were considered to have primary extragonadal NSGCT.

Testicular maldescent was documented in 35 of 442 (7.9%) records, unilateral in 25 and bilateral in ten. Ten NSGCTs arose in an uncorrected maldescended testis, 20 in a testis after orchidopexy, four in the normally descended contralateral testis and in two the diagnosis was of extragonadal NSGCT. Of the eight men who developed a second testicular cancer, only one had maldescent – bilateral and uncorrected. A prior diagnosis of inguinal hernia was documented in 15 records (3.4%). In few case records was there comment about absence of either testicular maldescent or inguinal hernia. Twenty-one men (4.8%) were recorded as having undergone vasectomy, the absence of this operation was noted only once. The date of vasectomy was documented for 15, in whom the median interval from vasectomy to diagnosis of NSGCT was 18 months (quartiles 8 and 38, range 5–100 months); five developed NSGCT within a year of vasectomy.

At presentation two men gave a family history of a testicular tumour, no negative family histories were recorded. A later note was made of five first- or second-degree relatives with the same diagnosis. In all, five brothers of four patients and three first cousin pairs were affected. Two of four sibling and one of three cousin pairs were registered by the West of Scotland Cancer Registry but only one cousin pair was treated at the same centre. There was no indication of the numbers of first- or second-degree male relatives at risk in these families or in the families of other cases.

Assigning men to a Carstairs and Morris deprivation category on the basis of their residential postcode confirmed the inverse association between age-standardised NSGCT incidence and deprivation (Table II).

The incidence of NSGCT in the west of Scotland increased during the 15 years 1975–1989 (Figure 1). Regression analyses predict that the overall and 15–44 year age-specific incidence will double in 20 and 25 years respectively. Statistically significant rises in incidence have occurred amongst men from more deprived areas (deprivation categories 3–7). No increase is apparent among residents of the more affluent areas (Table III).

**Table I** Reasons for non-inclusion of 44 men in the computerised West of Scotland Cancer Registry listing for teratoma

| | | | *Pathology* | | |
|---|---|---|---|---|---|
| | *Unexplained* | *ISD* | *Seminoma* | *Carcinoma* | *Site* |
| 1975–79 | 4 | 2 | 4 | 5 | – |
| 1980–84 | 1 | 6 | 4 | 5 | 4 |
| 1985–89 | 4 | – | 2 | 2 | – |
| Total | 9 | 8 | 10 | 12 | 4 |

ISD, The Information Services Division of the Common Services Agency, which was responsible for the take-on of pre-1985 manual registry data to the computerised system. There was one death certificate-only registration, in 1975

### Discussion

The estimate of non-registration (2–3.4%) for adult male NSGCT during the 15 years 1975–89 is identical to that for childhood cancers between 1971 and 1984 (Hawkins and Swerdlow, 1992). This compares well with the published literature, in which non-registration of between 2% and 28% is reported for adult cancers, depending on the tumour type (Nwene and Smith, 1982; Mukherjee *et al.*, 1991). Although inter-registry comparisons are rare, completeness of lymphoma registration is estimated to vary from 87% to 96% (Swerdlow *et al.*, 1993). The mechanism that permitted erroneous registration of prevalent tumours (11 of 426, 2.6%

**Table II** The relationship between socioeconomic status and incidence of non-seminomatous germ cell tumours in the West of Scotland 1975–89

| *Deprivation category (Carstairs and Morris)* | *Number of Cases* | | *Standardised incidence ratio* | *95% confidence interval* |
|---|---|---|---|---|
| | *Observed* | *Expected* | | |
| 1 (affluent) | 33 | 22.9 | 144 | 99–202 |
| 2 | 52 | 38.9 | 134 | 100–175 |
| 3 | 83 | 79.8 | 104 | 83–129 |
| 4 | 84 | 95.4 | 88 | 70–109 |
| 5 | 78 | 84.2 | 93 | 73–116 |
| 6 | 82 | 72.2 | 114 | 90–141 |
| 7 (deprived) | 30 | 43.9 | 68 | 46–98 |

$\chi^2$ for trend $P = 0.03$.

**Figure 1** Change in the incidence of teratoma, West of Scotland, 1975–89 —■—, All ages; +, 15–44 years.

in this study) is unclear, but of concern; and the extent to which this occurs can only be determined from comparison with the clinical record.

There has been a consistent, worldwide, post-war increase in testicular cancer incidence. In Scotland this increase predominantly affected NSGCT (Boyle *et al.*, 1987) in contrast the the similar rise in both NSGCT and seminoma documented from the USA (Schottenfeld *et al.*, 1980), Denmark (Osterlind, 1986) and Australia (Stone *et al.*, 1991). Our data, based on as complete and accurate case ascertainment as possible, confirm that the incidence of NSGCT in the West of Scotland is rising and likely to double in 20–25 years (Figure 1).

Reasons for this increase remain largely unexplained. The frequency of surgical intervention for testicular maldescent in the UK has doubled between 1962 and 1981 (Chilvers *et al.*, 1984). This is assumed to reflect a similar increase in incidence, but may represent an increase in surveillance and hence diagnosis, or intervention. Among this west of Scotland cohort, nearly one-third of cases with maldescent (11 of 35) had not undergone any attempt at surgical correction, so the potential for increasing intervention exists. However, only a minority (<13%) of cases are associated with maldescent (Pottern *et al.*, 1985; Swerdlow *et al.*, 1987; Strader *et al.*, 1988). Recent UK data suggest that lack of the protective effect of exercise and earlier puberty may be contributory (UK Testicular Cancer Study Group, 1994).

There is concern that vasectomy may be a risk factor for testicular cancer, in view of the parallel increase in both, and circumstantial supportive evidence (Thornhill *et al.*, 1988; Cale *et al.*, 1990). However, one study attributed the observation to under-reporting of vasectomy in Roman Catholic controls (Strader *et al.*, 1988), and more recent data fail to support the hypothesis (Neinhuis *et al.*, 1992; UK Testicular Cancer Study Group, 1994). An interval of less than 1 year between vasectomy and diagnosis in one-third of men in the west of Scotland who reported prior vasectomy, suggests reporting bias in an attempt to explain testicular swelling.

**Table III** Changes in incidence (average annual rate per 100 000) of NSGCT over time, by deprivation category

| *Deprivation category (Carstairs and Morris)* | *Time period* 1975–79 | 1980–84 | 1985–89 | *Test for trend* t | P |
|---|---|---|---|---|---|
| 1 + 2 | 2.6 | 3.3 | 3.0 | 0.56 | NS |
| 3 + 4 + 5 | 1.2 | 2.3 | 2.4 | 3.60 | <0.01 |
| 6 + 7 | 1.6 | 1.7 | 3.0 | 2.73 | 0.02 |

Neither clinical nor epidemiological publications have, until recently, suggested that testicular cancer may have a familial tendency. The literature includes anecdotal reports of 134 pairs of first-degree relatives (quoted from Forman *et al.*, 1992). Our observation that none of the pairs of first-degree relatives were treated at the same centre may contribute to lack of recognition of a familial association. Data from the UK case–control study indicate that the cumulative risk for brothers of cases in their first 50 years is 2.2% (Forman *et al.*, 1992), a figure similar to the crude risk of a second testicular cancer (1.8% of all cases and 2.6% of survivors in this study).

The incidence of cancer in Scotland is highest among residents of the most deprived postcode sectors, although a few types of cancer are more common in the affluent residential postcode areas (Carstairs and Morris, 1991); NSGCT incidence in the west of Scotland is in this latter category. East Anglia data suggested that seminoma rather than NSGCT incidence was related to social class (Nethersall and Sikora 1984), although this observation was based on fewer patients and occupationally derived social class was not available for all cases.

Our data further suggest that the observed increase in NSGCT incidence is confined to men resident in more deprived areas; indeed by 1985–89, no socioeconomic gradient is apparent (Table III). Additional studies are required to confirm this finding. It is possible that, if this trend continues, NSGCT will no longer be associated with higher socioeconomic status, and may even become a deprivation-related tumour.

### References

BOYLE P, KAYE SB AND ROBERTSON AG. (1987). Changes in testicular cancer in Scotland. *Eur. J. Cancer Clin. Oncol.*, **23**, 827–830.

CALE ARJ, FAROUK M, PRESCOTT RJ AND WALLACE IWJ. (1990). Does vasectomy accelerate testicular tumour? Importance of testicular examinations before and after vasectomy. *Br. Med. J.*, **300**, 370.

CARSTAIRS V AND MORRIS R. (1991). *Deprivation and Health in Scotland.* Aberdeen University Press: Aberdeen, UK.

CHILVERS CED, PIKE MC, FORMAN D, FOGELMAN K AND WADSWORTH MEJ. (1984). Apparent doubling of frequency of undescended testis in England and Wales 1962–1981. *Lancet*, **2**, 330–332.

FORMAN D, OLIVER RTD, BRETT AR, MARSH, SGE, MOSES JH, BODMER JG, CHILVERS CED AND PIKE MC. (1992). Familial testicular cancer: a report of the UK family register, estimation of risk, and an HLA Class I sib-pair analysis. *Br. J. Cancer*, **65**, 255–262.

HAWKINS MM AND SWERDLOW AJ. (1992). Completeness of cancer and death follow-up obtained through the National Health Service Central Register for England and Wales. *Br. J. Cancer*, **66**, 408–413.

HENDERSON BE, BENTON B, JING J, YU MC AND PIKE MC. (1979). Risk factors for cancer of the testis in young men. *Int. J. Cancer*, **23**, 598–602.

MORRIS BROWN L, POTTERN LM AND HOOVER RN. (1987). Testicular cancer in young men: the search for causes of the epidemic increase in the United States. *J. Epidemiol. Community Health*, **41**, 349–354.

MUKHERJEE AK, LECK I, LANGLEY FA AND ASHCROFT C. (1991). The completeness and accuracy of health authority and cancer registry records according to a study of ovarian neoplasms. *Public Health*, **105**, 69–78.

NEINHUIS H, GOLDACRE M, SEAGROATT V, GILL L AND VESSEY M. (1992). Incidence of disease after vasectomy: a record linkage retrospective cohort study. *Br. Med. J.*, **304**, 743–746.

NETHERSELL ABW AND SIKORA K. (1984). Testicular cancer and social class in East Anglia. *Br. J. Cancer*, **50**, 537–540.

NWENE U AND SMITH A. (1982). Assessing completeness of cancer registration in the North-Western Region of England by a method of independent comparison. *Br. J. Cancer*, **46**, 635–639.

OSTERLIND O. (1986). Diverging trends in incidence and mortality of testicular cancer in Denmark, 1943–1982. *Br. J. Cancer*, **53**, 501–505.

PEARCE N, SHEPPARD RA, HOWARD JK, FRASER J AND LILLEY BM. (1987). Time trends and occupational differences in cancer of the testis in New Zealand. *Cancer*, **59**, 1677–1682.
PIKE MC, CHILVERS C AND PECKHAM MJ. (1986). Effect of age at orchidopexy on risk of testicular cancer. *Lancet*, **1**, 1246–1248.
POTTERN LM, MORRIS BROWN L, HOOVER RN, JAVADPOUR N, O'CONNELL KJ, STUTZMAN RE AND BLATTNER WA. (1985). Testicular cancer risk among young men: role of cryptorchidism and inguinal hernia. *J. Natl. Cancer Inst.*, **74**, 377–381.
SCHOTTENFELD D, WARSHAUER ME, SHERLOCK S, ZAUBER AG, LEDER M AND PAYNE R. (1980). The epidemiology of testicular cancer in young adults. *Am. J. Epidemiol.*, **112**, 232–246.
STONE JM, CRUICKSHANK DG, SANDEMAN TF AND MATTHEWS JP. (1991). Trebling of the incidence of testicular cancer in Victoria, Australia (1950–1985). *Cancer*, **68**, 211–219.
STRADER CH, WEISS NS AND DALING JR. (1988). Vasectomy and the incidence of testicular cancer. *Am. J. Epidemiol.*, **128**, 56–63.
SWERDLOW AJ AND SKEET RG. (1988). Occupational associations of testicular cancer in south east England. *Br. J. Ind. Med.*, **45**, 225–230.
SWERDLOW AJ, HUTTLY SRA AND SMITH PG. (1987). Testicular cancer and antecedent diseases. *Br. J. Cancer*, **55**, 97–103.
SWERDLOW AJ, DOUGLAS AJ, VAUGHAN HUDSON G AND VAUGHAN HUDSON B. (1993). Completeness of cancer registration in England and Wales: an assessment based on 2,145 patients with Hodgkin's disease independently registered by the British National Lymphoma Investigation. *Br. J. Cancer*, **67**, 326–329.
THORNHILL JA, CONROY RM, KELLY DG, WALSH A, FENNELLY JJ AND FITZPATRICK JM. (1988). An evaluation of predisposing factors for testis cancer in Ireland. *Eur. Urol.*, **14**, 429–433.
UNITED KINGDOM TESTICULAR CANCER STUDY GROUP. (1994). Aetiology of testicular cancer: association with congenital abnormalities, age at puberty, infertility and exercise. *Br. Med. J.*, **308**, 1393–1399.